# Supplementary material for: Low frequency oscillations – neural correlates of stability and flexibility in cognition
Source: Nat Commun. 2025 Jun 25;16:5381. doi: 10.1038/s41467-025-60821-2 (PMC12198418; doi:10.1038/s41467-025-60821-2)
Supplement: Supplementary file 1 — Supplementary Information [file 41467_2025_60821_MOESM1_ESM.pdf]

Supplementary information

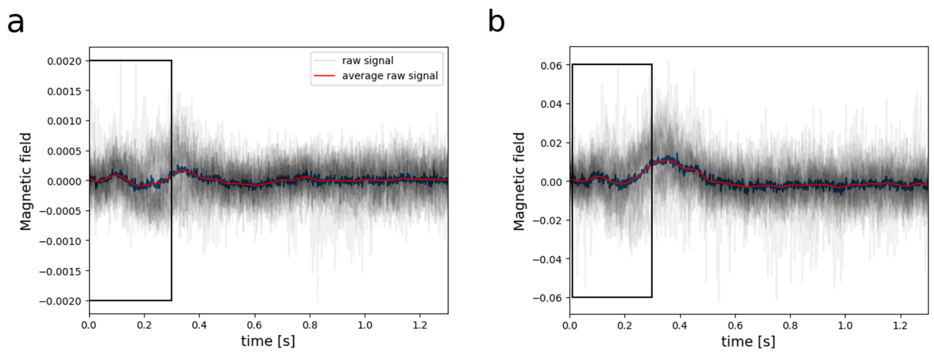

Supplementary Fig. 1: The oscillations during encoding, phase-locked to the onset of the stimulus. Difference between the posterior signal and the global signal. *a) An example of MEG-measured activtiy during presentation and delay in the Odd One Out task from the posterior network. The raw signals in grey, the averaged signal in blue, and the smoothed average signal in red. The box marks the presenation period, where we see posteior theta oscillations. b) The same signals but averaged over the whole cortex instead of only the posterior areas. Importantly, the posterior network show much clearer theta oscillations compared to the whole-brain averaged activtiy.*

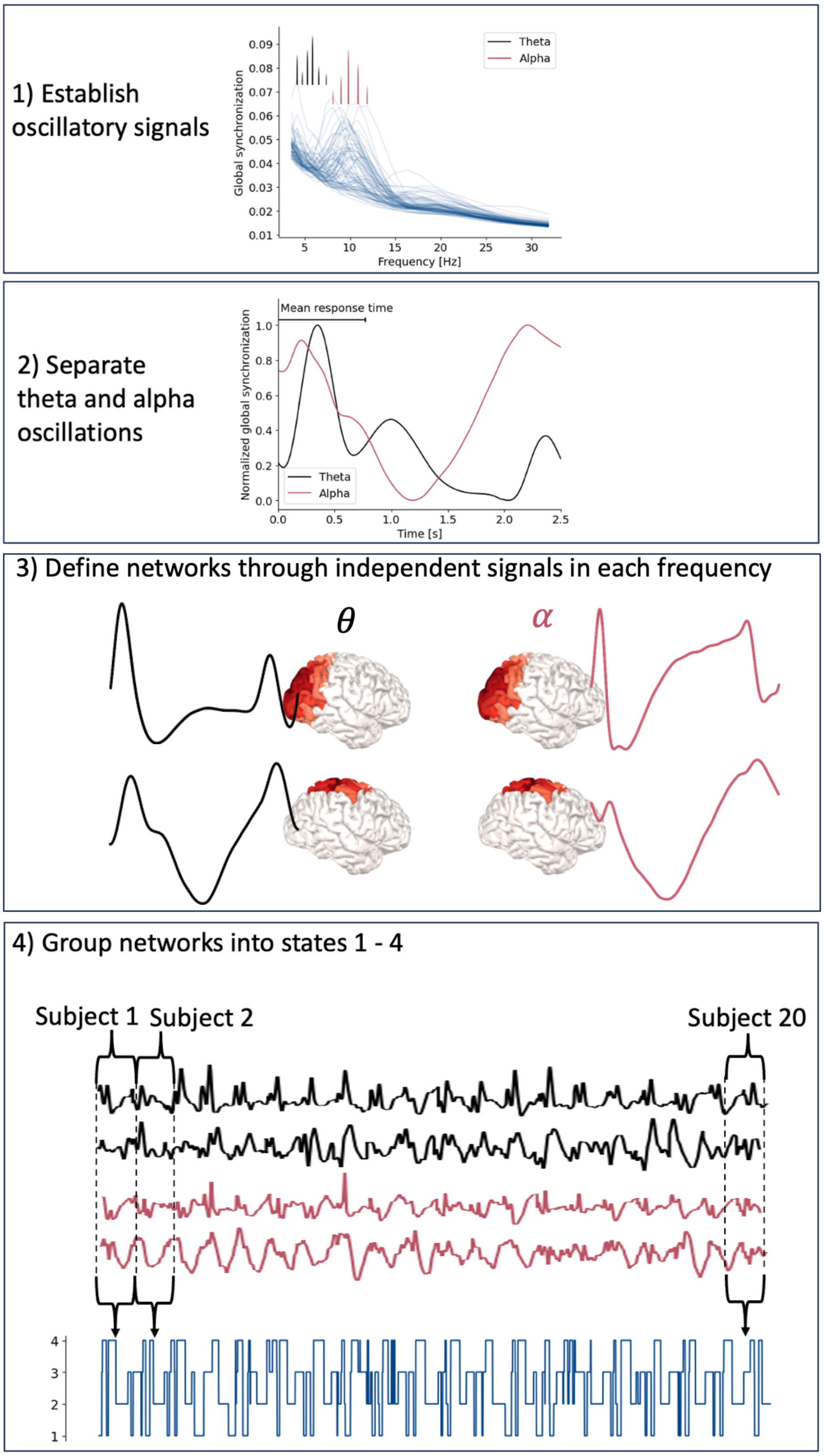

Supplementary Fig. 2: The four steps in the state creation pipeline*. 1) We establish the mean theta and alpha peak frequencies in the synchronization spectras. 2) Using Morlet filtering, we separate the alpha and theta activities in the data using the meak peak frequencies. 3) We identify independent components within each of the two wavelets. 4) We concatenate the signals generated by the ICA to cluster each time point of the trial into one of four states.*

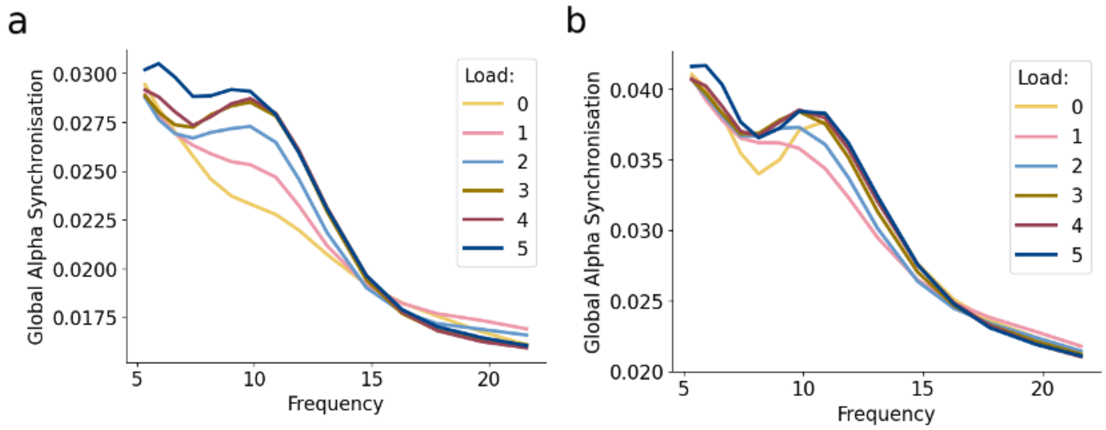

Supplementary Fig. 3: The global synchronization for WM loads 0 – 5 averaged over participants and sessions, *for a) WM-Grid and b) Odd One Out.*

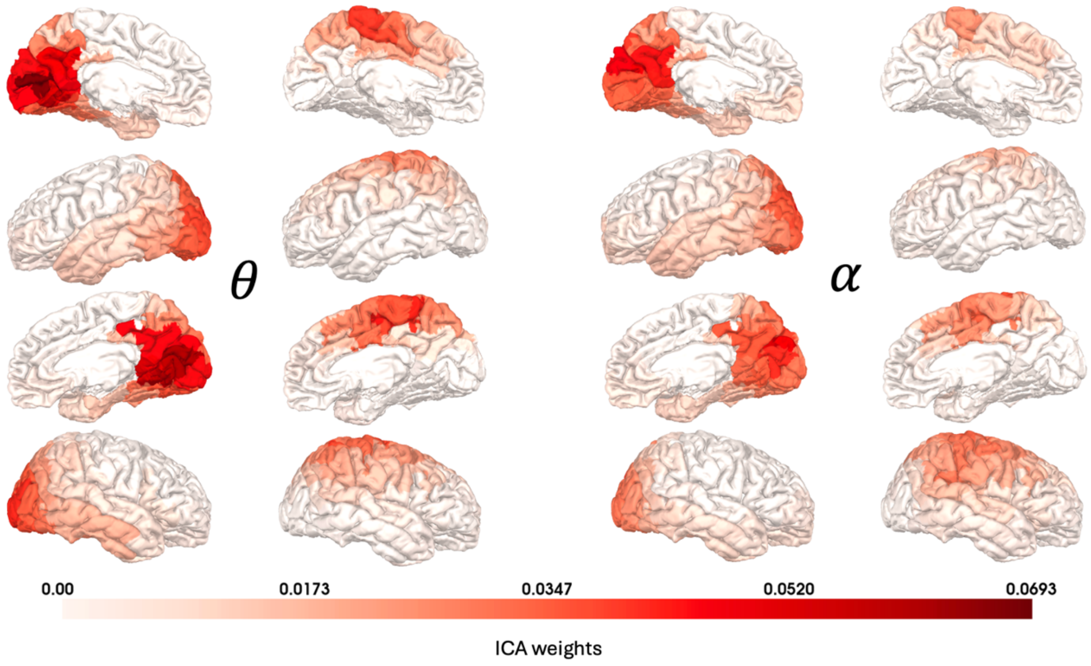

Supplementary Fig. 4: The networks for the distractor dataset averaged across the 13 subjects. *Source data are provided as a Source Data file.*

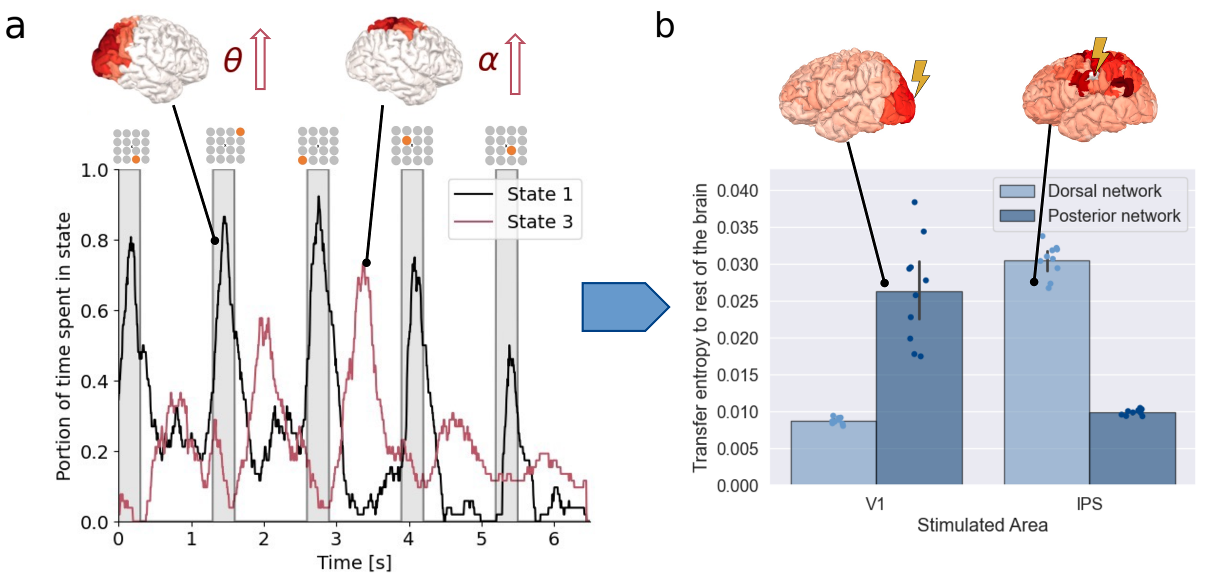

Supplementary Fig. 5: A schematic overview of main findings. *a) Large-scale oscillatory networks in the theta and alpha bands are systematically formed under specifc periods of cognitve tasks. Specifically, posterior theta oscillations (state 1) increase during vsWM encoding and dorsal alpha oscillations (state 3) increase during vsWM maintenance. b) Our simulations show that these networks could be used to dynamically route information to meet the current task demands. For example, in the simulations, the posterior theta network increased information transfer between visual and parietal areas which can be used for encoding. The dorsal alpha network instead increased information tranfer between frontoparietal areas, important for vsWM maintenance.*

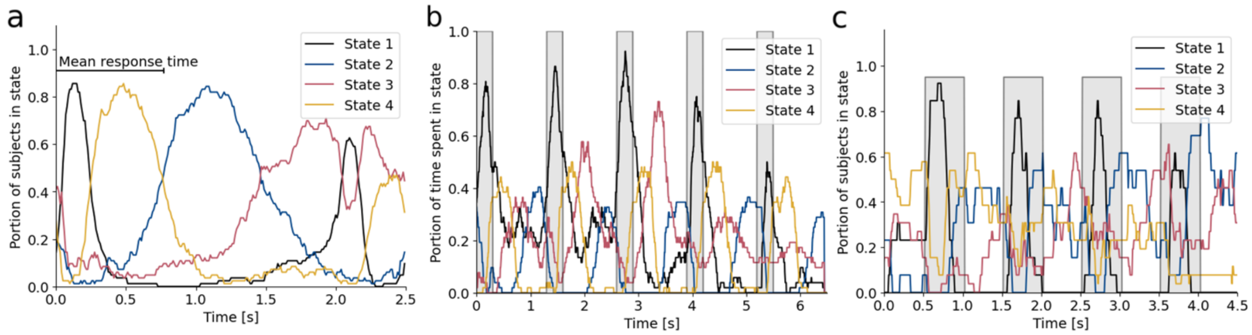

Supplementary Fig. 6: The complete state time *series for a) the Human Connectome Project dataset, b) the 4-subject dataset, and c) the distractor dataset*. Source data are provided as a Source Data file.
