# Supplementary material for: Low frequency oscillations – neural correlates of stability and flexibility in cognition
Source: Nat Commun. 2025 Jun 25;16:5381. doi: 10.1038/s41467-025-60821-2 (PMC12198418; doi:10.1038/s41467-025-60821-2)
Supplement: Supplementary file 2 — Reporting Summary [file 41467_2025_60821_MOESM2_ESM.pdf]

nature portfolio

Corresponding author(s): Torkel Klingberg

Last updated by author(s): Jan 8, 2025

Reporting Summary

Nature Portfolio wishes to improve the reproducibility of the work that we publish. This form provides structure for consistency and transparency in reporting. For further information on Nature Portfolio policies, see our Editorial Policies and the Editorial Policy Checklist.

Statistics

For all statistical analyses, confirm that the following items are present in the figure legend, table legend, main text, or Methods section.

n/a	Confirmed	
☐	☒	The exact sample size (*n*) for each experimental group/condition, given as a discrete number and unit of measurement
☐	☒	A statement on whether measurements were taken from distinct samples or whether the same sample was measured repeatedly
☐	☒	The statistical test(s) used AND whether they are one- or two-sided<br>*Only common tests should be described solely by name; describe more complex techniques in the Methods section.*
☒	☐	A description of all covariates tested
☐	☒	A description of any assumptions or corrections, such as tests of normality and adjustment for multiple comparisons
☐	☒	A full description of the statistical parameters including central tendency (e.g. means) or other basic estimates (e.g. regression coefficient) AND variation (e.g. standard deviation) or associated estimates of uncertainty (e.g. confidence intervals)
☐	☒	For null hypothesis testing, the test statistic (e.g. *F*, *t*, *r*) with confidence intervals, effect sizes, degrees of freedom and *P* value noted<br>*Give P values as exact values whenever suitable.*
☒	☐	For Bayesian analysis, information on the choice of priors and Markov chain Monte Carlo settings
☒	☐	For hierarchical and complex designs, identification of the appropriate level for tests and full reporting of outcomes
☐	☒	Estimates of effect sizes (e.g. Cohen's *d*, Pearson's *r*), indicating how they were calculated

Our web collection on statistics for biologists contains articles on many of the points above.

Software and code

Policy information about availability of computer code

Data collection	MEG data was acquired with a 306-channel whole-head MEG system (Elekta Neuromag TRIUX) and T1-weighted structural MRIs (1x1m2) were aquired with a 3 Tesla scanner (GE SIGNA Premier).
Data analysis	Preprocessing of MEG and MRI data was performed with FreeSurfer and MaxFilter for tSSS. All subsequent data analysis was performed in Python 3.9. We used several packages for the analysis in python: jupyter 1.0, matplotlib 3.8, mne 1.6, numpy 1.26, pandas 2.2, QtPy 2.4, scipy 1.12 and seaborn 1.8. Code for simulations can be found at https://github.com/juliaericson/Information-Flow-In-Silico-Brain.

For manuscripts utilizing custom algorithms or software that are central to the research but not yet described in published literature, software must be made available to editors and reviewers. We strongly encourage code deposition in a community repository (e.g. GitHub). See the Nature Portfolio guidelines for submitting code & software for further information.

Data

Policy information about availability of data

All manuscripts must include a data availability statement. This statement should provide the following information, where applicable:

- Accession codes, unique identifiers, or web links for publicly available datasets
- A description of any restrictions on data availability
- For clinical datasets or third party data, please ensure that the statement adheres to our policy

Raw MEG and MRI data from the Human Connectome Project is available at https://www.humanconnectome.org/. Structural data for simulations are from the

MICA-MICs dataset (https://portal.conp.ca/dataset?id=projects/mica-mics). Data from the distractor dataset is available at https://osf.io/gu25f/?view_only=. Data from the 4-subject dataset is available at https://osf.io/8mvwy/.

Research involving human participants, their data, or biological material

Policy information about studies with human participants or human data. See also policy information about sex, gender (identity/presentation), and sexual orientation and race, ethnicity and racism.

Reporting on sex and gender	Gender was reported but no further sex or gender based analyses were performed as that was beyond the scope of this study.
Reporting on race, ethnicity, or other socially relevant groupings	We did not collect/have access to reporting on race and ethnicity.
Population characteristics	Young adults
Recruitment	Participants for the 4-subject dataset were recruited using an ad on the Karolinska Institutet website.
Ethics oversight	Swedish Ethics Committee

Note that full information on the approval of the study protocol must also be provided in the manuscript.

Field-specific reporting

Please select the one below that is the best fit for your research. If you are not sure, read the appropriate sections before making your selection.

☐ Life sciences ☒ Behavioural & social sciences ☐ Ecological, evolutionary & environmental sciences

For a reference copy of the document with all sections, see nature.com/documents/nr-reporting-summary-flat.pdf

Behavioural & social sciences study design

All studies must disclose on these points even when the disclosure is negative.

Study description	4-subject dataset: Small N with dense sampling design<br>Human Connectome Project: Quantitative cross-sectional<br>Distractor dataset: Quantitative cross-sectional
Research sample	4-subject dataset: Young adults age 21, 21, 22 and 26. All from Sweden . Two men and two women (self-reported). Young adults were chosen because we looked at effects of training and wanted young subjects with higher plasticity. However, we also wanted subjects above 18 for practical and ethical reasons.<br>Human Connectome Project: 83 young adults (45 men, self-reported), age 22 -35.<br>Distractor dataset: 17 participants (10 men), age 21 - 41 from Sweden.
Sampling strategy	4-subject dataset: The participants followed a strict schedule which required all participants to be tested on the same days. This limited the number of participants to 5 subjects (each test session was 2 hours).<br>Human Connectome Project: N/A (preexisting dataset)<br>Distractor dataset: N/A (preexisting dataset)
Data collection	4-subject dataset: MEG and structural MRI data was collected. Apart from the participant the researcher and a lab assistant was present at data collection. For MRI, a lab technician was also present. The researcher was not blind to the study hypothesis during data collection.<br>Human Connectome Project: N/a (preexisting dataset)<br>Distractor dataset: N/A (preexisting dataset)
Timing	4-subject dataset: Data collected between 8 feb 2022 and 31 march 2022<br>Human Connectome Project: Data collected between 2012 and 2015<br>Distractor dataset: Data collected between May 2022 and May 2023
Data exclusions	4-subject dataset: No exclusions<br>Human Connectome Project: No known exclusions<br>The distractor dataset: One man was excluded due to magnetic inference and another man was excluded due to excessive movements resulting in the 17 subjects that were then made available. 4 subjects were also removed due to the inability to find networks in the theta band. This resulted in a total of 13 participants.
Non-participation	4-subject dataset: A fifth subject was included but dropped out after the first session<br>Human Connectome Project: Not known<br>The distractor dataset: Not known

Randomization	Not allocated into experimental groups. This applies for all datasets

Reporting for specific materials, systems and methods

We require information from authors about some types of materials, experimental systems and methods used in many studies. Here, indicate whether each material, system or method listed is relevant to your study. If you are not sure if a list item applies to your research, read the appropriate section before selecting a response.

Materials & experimental systems

n/a	Involved in the study
☒	☐ Antibodies
☒	☐ Eukaryotic cell lines
☒	☐ Palaeontology and archaeology
☒	☐ Animals and other organisms
☒	☐ Clinical data
☒	☐ Dual use research of concern
☒	☐ Plants

Methods

n/a	Involved in the study
☒	☐ ChIP-seq
☒	☐ Flow cytometry
☒	☐ MRI-based neuroimaging

Plants

Seed stocks	N/A
Novel plant genotypes	N/A
Authentication	N/A
